# Efficacy evaluation of lotilaner (Credelio™) in experimentally induced *Otodectes cynotis* infestations in cats

**DOI:** 10.1186/s13071-025-07081-7

**Published:** 2025-10-29

**Authors:** Brad Hayes, Alta Viljoen, Sofia Rouenhoff, Shubhi Mehrotra, Susan Michler, Liisa Ahlstrom, Friederike Krämer, Bettina Schunack

**Affiliations:** 1 Elanco Animal Health UK Ltd, Hook, UK; 2 Clinvet, Bloemfontein, South Africa; 3 Elanco Animal Health GmbH, Monheim, Germany; 4 Elanco Innovation and Alliance Centre (IAC), Bangalore, India; 5 Elanco Australasia Pty Ltd., Macquarie Park, New South Wales Australia; 6 TransMIT GmbH, Giessen, Germany

**Keywords:** Isoxazolines, Ectoparasiticide, Ear mite, Otocariosis, Otodectic mange, *Otodectes cynotis*, Credelio™, Lotilaner, Cat, Efficacy

## Abstract

**Background:**

The ear mite, *Otodectes cynotis*, is pathogenic, highly contagious and a global cause of otitis externa and pruritus in cats and dogs. The present study evaluated the efficacy of a single oral application of lotilaner flavoured chewable tablets for cats (Credelio™; Elanco, Greenfield, IN, USA) in cats experimentally infested with *O. cynotis*.

**Methods:**

Sixteen adult cats were experimentally infested with *O. cynotis* and confirmed to be mite positive by otoscopic examination. Infested cats were randomly assigned to one of two study groups. On day 0, a group of eight cats was treated once with Credelio™ at 7.0–11.8 mg lotilaner/kg body weight (i.e. at the lower end of the recommended dose range [6–24 mg/kg]), while in the control group eight cats were sham-dosed. All cats were dosed in a fed state. Otoscopic examinations for scoring the number of live mites and the amount of debris/cerumen were performed post treatment (p.t) on days 14 and 28. Ear flushing and microscopic viable mite counts were performed on day 28.

**Results:**

A single oral dose of Credelio™ on day 0 resulted in a 99.6% reduction in *O. cynotis* geometric mean mite counts in the Credelio™-treated study group on day 28 recovered by ear flushing. Otoscopic live ear mite count scores on days 14 and 28 did not detect any mites in the Credelio™-treated group. In contrast to this 100% improvement, in the control group an improvement of otoscopic live mite scores was recorded in 25% (day 14) and 12.5% (day 28) of the cats, respectively. Additionally, a comparison of the debris scores to baseline data on day − 2 showed an improvement in 75% of the Credelio™-treated cats on day 14 and in 87.5% on day 28 and no or limited improvement (0% on day 14 and 12.5% on day 28) in the control group animals.

**Conclusions:**

The results of this study demonstrated that a single application of Credelio™ chewable tablets for cats at the lower end of the recommended dose range was highly efficacious (99.6%) in eliminating experimentally induced *O. cynotis* infestations and greatly improved clinical signs of otocariosis in cats by 28 days p.t.

**Graphical Abstract:**

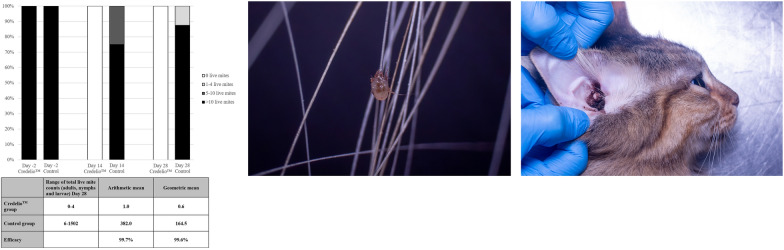

## Background

The ear mite *Otodectes cynotis* is a non-burrowing, obligate surface-living mite from the family Psoroptidae. *Otodectes cynotis* is not species-specific and may affect cats, dogs and other mammals [1]. The mite causes a parasitic disease of the external ear canal, classified as otodectic mange or otocariosis.

Depending on the cat population and the sensitivity of the diagnostic methods used, prevalence estimates on ear mite infestations in cats may vary widely [2]. Prevalence studies in mainly feral cat populations reported up to > 29–37% *O. cynotis*-positive animals [2–5], while up to 47.1–66.1% ear mite-positive animals were reported when the population included only otitis patients [2, 6, 7]. In a European survey, ear mites were found to be the most common ectoparasite in cats, even more commonly recovered than fleas [8]. Generally, *O. cynotis* mites are reported worldwide [9].

Adult mites are white and large (females: 345–451 μm; males: 274–362 μm) and move freely [10, 11]. The entire life-cycle (egg, larva, nymph, adult) takes place in the ear canal of the host [9, 12] and requires approximately 18–28 days [10, 13]. Adult mites survive on the host for approximately 2 months [1] and can survive for up to 12 days off the host in ideal temperature conditions [14]. Mites feed on epidermal debris and tissue fluids from the superficial epidermis, irritating the epithelium and stimulating the production of large amounts of ear cerumen, occasionally mixed with blood, which fills the ear canal [11, 12]. Otodectic mange causes pruritic, erythematous and ceruminous otitis externa, and almost always occurring bilaterally. Otodectic otitis is characterized by large amounts of brown-black dry cerumen, resembling “coffee powder” [12]. However, secondary bacterial or yeast infections are possible [15], and the character of the exudate may change quickly in the presence of these secondary infections [16]. In addition to the classical otitis externa, ‘ectopic’ infestations of the head, neck, tail head and—rarely—the trunk can occur when mites escape the ear canals [17], and papulocrustous lesions (miliary dermatitis) may be observed [18]. In cats, the disease is highly variable, and the severity of signs does not necessarily correlate with the number of mites present on the host [10]. Affected cats can present in a wide range of states, varying from apparently healthy [19] to severe otitis externa [9]. Cats can develop hypersensitivity reactions to *O. cynotis*, and small numbers of mites in these cats can still be associated with clinical signs [20], with affected hypersensitive cats showing severe pruritus that is not proportionate to the number of mites in the ear canal [21]. On the contrary, some cats may have huge numbers of mites in the external ear canal without pruritus, which is possibly explained by the absence of hypersensitivity phenomena [10]. Pruritus severity is responsible for auto-traumatic lesions, such as alopecia, erosions, ulcers and crusting affecting the preauricular regions, head, face and neck, and for otohematomas [9].

The disease is extremely contagious, and transmission occurs primarily by direct contact with infested cats. Infestation of one ear from the other in the same cat is also common [19]. The mites may easily spread in multi-cat and multi-pet environments. A temporary infestation of humans may occur [22, 23], underlining the potential zoonotic character of the disease. Otodectic mange affects kittens and adults, but juveniles are predisposed [19].

The history and clinical signs are highly suggestive of *Otodectes* infestation. Careful otoscopic examination often reveals the mites as they move and confirms the diagnosis. Additionally, *Otodectes* mites can be seen on microscopic examination of the waxy debris broken up in liquid paraffin [20].

In the study reported here, lotilaner was evaluated for the treatment of otodectic mange. Lotilaner (Credelio™, Elanco; Elanco, Greenfield, IN, USA) is the most recent addition to the novel class of ectoparasiticidal isoxazolines, and is registered for monthly administration to cats and dogs. With a peak blood concentration after administration (T_MAX_) recorded at 4 hours and a terminal half-life of 33.6 days (in the fed condition), lotilaner provides quick, sustained, consistent efficacy against flea and tick infestations for 1 month in cats [24–26]. Studies in both cats and dogs have demonstrated lotilaner’s safety and its fast, sustained efficacy against a range of feline and canine ectoparasites, including several tick species (*Amblyomma americanum*, *Amblyomma cajennense*, *Dermacentor reticulatus*, *Dermacentor variabilis*, *Haemaphysalis longicornis*, *Ixodes hexagonus*, *Ixodes holocyclus*, *Ixodes ricinus, Ixodes scapularis* and *Rhipicephalus sanguineus*) [26–33], fleas (*Ctenocephalides felis* and* Ctenocephalides canis*) [25, 34–41] and mites (*Demodex* spp.) [42].

Until the development of the isoxazolines, the treatment of mites in cats and dogs, including *O. cynotis*, relied mostly upon topically applied endectocides containing a macrocyclic lactone (moxidectin [43, 44] or selamectin [45, 46]). While recent studies have demonstrated the excellent efficacy of the isoxazoline class against mites [47–52], there are no published studies evaluating the efficacy of orally administered lotilaner in cats for the control of *O. cynotis*. The objective of this study was to evaluate the efficacy of a single dose of lotilaner (Credelio™ chewable tablets for cats) for the treatment of experimentally induced *O. cynotis* infestations in cats under laboratory conditions.

## Methods

### Study design

The study was conducted in South Africa in accordance with the standards of Good Clinical Practice (Veterinary International Conference on Harmonization [VICH] Guideline 9) and the Efficacy of Ectoparasiticides Guideline (7AE17a) [53, 54], following the guidance on the size of treatment groups from the latest Committee for Veterinary Medicinal Products (CVMP) guideline [55]. The study was a parallel group, blinded, randomized, singlecentre, negatively controlled, efficacy study with the aim to evaluate the effectiveness of lotilaner in the investigational veterinary product (IVP) Credelio™ chewable tablets for cats for the treatment of cats infested with *O. cynotis* under laboratory conditions, compared to a sham-dosed control group. Blinding of the study personnel was enabled by separation of study roles. On day 0, the cats in the IVP-treated group (*n* = 8 cats) were dosed with Credelio™ chewable tablets for cats according to label instructions, in a fed state at the lower end of the recommended dose range, and the control group (*n* = 8 cats) was sham-dosed.

### Animal details

Cats included into the study were crossbreeds of both sexes (5 males and 3 females per group), aged between 1.3 and 7.3 years old and weighing between 2.76 and 5.92 kg (day −2).

Cats were experimentally infested approximately 1 month prior to study start with 80–100 ear mites into each ear. *Otodectes cynotis* infestations were established by harvesting mites by lavage from donor animals and transferring them into each ear of recipient animals. After a general clinical examination and the confirmation of ear mite infestation by means of otoscopic examination on day –7, cats were enrolled into the study and underwent a 7-day acclimatization period. On day –2, ear mite infestation was again confirmed by otoscopic examination, and all animals were further confirmed to be healthy upon physical examination prior to treatment, apart from otic signs of ear mite infestation.

For the duration of the study, all cats were housed individually in appropriately sized cages with visual and auditory but no physical contact between the animals. Each cage had an elevated sleeping platform and scratch post. At least one toy was provided to each cat and changed weekly. All cats were fed once daily with a standard, commercially available dry food for cats, according to the manufacturer’s recommendation, and had free access to potable municipal water.

None of the cats included into the study had been treated with drugs which could interfere with the objectives of the study prior to the study start.

For group randomization, all study animals were ranked within each sex based on individual pre-treatment mite count scores (day −2). Female cats were ranked first by decreasing mite count scores, followed by males ranked identically. Subsequently ranked animals were blocked into eight blocks of two cats each. Ascending order of animal identification ID were used to break ties. Within blocks, cats were randomly allocated to the two study groups (Credelio™-treated group and sham-dosed control group).

### Animal health

All cats were deemed clinically healthy excluding signs of ear mite infestation upon physical examination on days −7 and −2. General health observations were performed once daily throughout the complete study duration. A final physical examination was performed by the attending veterinarian on day 28 at the end of the study.

### Treatment

On day 0, cats in the IVP group were fed approximately 1.5–1 h before being treated with Credelio™ chewable tablets for cats. Cats were dosed with one or two tablets to ensure dosing at the lower end of the recommended dose range (6–24 mg/kg), so that cats received a dose of lotilaner of between 7.0 and 11.8 mg/kg body weight. Doses were determined based on the individual body weights recorded on day −2 and the nominal content of lotilaner in the tablets. With the cats standing, the Credelio™ tablets were applied onto the back of each cat’s tongue. The cats were restrained for about 1 min to allow the product to be swallowed. Cats in the control group were handled and sham-dosed following the same procedure as cats in the Credelio™ group. Cats in both groups were clinically assessed before the treatment and again at 1 h (± 15 min), 2 h (± 15 min), 4 h (± 30 min), and 8 h (± 30 min) after treatment or sham-dosing.

### Assessment of ear mite infestation

An otoscopic examination of both ears from each cat was performed prior to animal enrollment on day −7, prior to treatment on day −2 for group randomization and on days 14 and 28 after treatment (in the latter case prior to ear flushing) to assess the number and viability of ear mites as well as amount of debris. Live mite scores were assigned as: 0 = 0 live mites, 1 = 1–4 live mites, 2 = 5–10 live mites or 3 =  > 10 live mites. For inclusion into the study, a cat was considered to be adequately infested if at least one ear had a mite score of 3 (> 10 viable mites) and the presence of live mites was at least confirmed in the other ear. On days 14 and 28, live mite scores were based on the total number of mites from both ears. Additionally, at the specified time points listed above, the amount of debris/cerumen within each ear canal was recorded during the otoscopic examination and scored as: 0 = no debris/cerumen, 1 = slight debris/cerumen, 2 = moderate debris/cerumen or 3 = severe debris/cerumen.

A quantitative assessment of viable mites was performed on all cats on day 28 under sedation by ear canal flushing. After sedation using ketamine (Anaket-V®; Bayer AG, Leverkusen, Germany) and medetomidine (Domitor®; Orion Corp., Espoo, Finland), subsequently reversed by atipamezole hydrochloride (Antisedan®, Zoetis, Parsippany-Troy Hills, NJ, USA), the ear canals were filled with a 5% aqueous solution of docusate sodium (DocuSol®; Kyron Laboratories [Pty] Ltd., Johannesburg, South Africa) and massaged lightly to loosen and soften the debris, following which the solution was collected from the ears and drained through a 38-μm sieve. The ears were then flushed with lukewarm saline solution, which was also collected and poured through the same sieve. The ears were examined otoscopically and, if needed, the flushing process was repeated until the ear canals were judged to be clean with no observed cerumen or mites. The sieved contents were rinsed with clean water and transferred to separate containers for the left and right ear. The contents of each container were examined under the microscope on the day of collection for number and integrity of mite stages (eggs, larvae, nymphs, adults). If viable mites were observed during the otoscopic examination, all intact mite stages retrieved after flushing of that specific ear were recorded as “live mites”. The counts from both ears were added up to give each cat’s total ear mite count.

### Efficacy evaluation and data analysis

The software package SAS® (version 9.4 TS Level 1 M2; SAS Institute Inc., Cary, NC, USA) was used for all the statistical analyses with the individual cat as experimental unit. For primary efficacy calculations, the total numbers of live mites (sum of adults, nymphs and larvae from both ears) counted following ear flushing on day 28 were used. The percentage of efficacy against *O. cynotis* mites was calculated as follows:

$${\text{Efficacy }}\left( \% \right) \, = {1}00{\text{x }}\left( {{\text{MC}}{-}{\text{MT}}} \right) \, /{\text{ MC}}$$

where M_C_ was the mean number of live mites in the control group and M_T_ the mean number of live mites in the Credelio™-treated group. The calculation was made using both geometric and arithmetic means.

The log-counts (count + 1) of live mites of the Credelio™ treated group were compared to the log-counts (count + 1) of the control group using a mixed analysis of variance model to confirm the miticidal efficacy results, including study group and sex as fixed factors and block as a random effect. All two-sided statistical tests had a 5% level of significance. In addition to linear mixed model analysis, the Wilcoxon-Mann-Whitney test was also used to compare between-group values. Efficacy was determined by ≥ 90% reduction of ear mites in the Credelio™ treated group and a statistically significant difference (*P* value ≤ 0.05) between the Credelio™ treated and the control group.

Secondary efficacy criteria were the improvement in debris score and the reduction in live mite score. Here, the treatment effect on the otoscopically assessed debris score and live mite count score was determined by comparing the worst-case score between the two ears at days 14 and 28 with the worst-case score between the two ears at baseline (day −2). A comparison between treatment groups was made using the Cochran-Mantel-Haenszel (CMH) test to obtain the row mean scores.

## Results

All cats had an adequate mite infestation at the commencement of the study (day −2), having a mite score of 3 (> 10 viable mites) in one ear and at least confirmed presence of ear mites in the other ear, with 13 of the 16 cats having a mite score of 3 in both ears.

The arithmetic mean number of live mites recovered from cats in the control group after ear flushing on day 28 was 382 (Table 1), confirming the presence of an adequate mite infestation throughout the study. Further confirmation was the presence on day 28 of at least 11 viable *O. cynotis* mites, across both ears, in seven out of eight cats (87.5%) in the control group.

**Table 1:** Individual and arithmetic mean mite count scores (assessed otoscopically on days −2, 14 and 28) and total mite numbers (sum of left and right ear, recovered and counted after ear flushing on day 28) in both treatment groups

Study group	Animal ID	Mite count score^a^ (worst case^b^)			Total mite numbers^b^ after ear flushing day 28				
		Day −2	Day 14	Day 28	Eggs	Larvae	Nymphs	Adults	Sum (excluding eggs)^c^
Credelio™ group	1	3	0	0	0	0	0	0	0
	2	3	0	0	0	0	0	0	0
	3	3	0	0	0	0	0	0	0
	4	3	0	0	0	0	0	0	0
	5	3	0	0	0	0	0	0	0
	6	3	0	0	0	0	0	4	4
	7	3	0	0	0	0	0	2	2
	8	3	0	0	0	2	0	0	2
	AM	3.0	0.0	0.0	0.0	0.3	0.0	0.8	1.0
Control group	9	3	3	3	13	6	68	96	170
	10	3	2	3	2	3	26	26	55
	11	3	3	3	2	12	32	93	137
	12	3	2	1	0	0	2	4	6
	13	3	3	3	0	0	105	182	287
	14	3	3	3	2	19	74	107	200
	15	3	3	3	11	92	130	477	699
	16	3	3	3	3	215	345	942	1502
	AM	3.0	2.8	2.8	4.1	43.4	97.8	240.9	382.0

AM arithmetic mean

^a^Score categories: 0 = 0 live mites; 1 = 1–4 live mites; 2 = 5–10 live mites; 3 =  > 10 live mites

^b^Of both ears

^c^Larvae, nymphs and adults of both ears; numbers used for efficacy calculation

The actual dose of lotilaner administered to cats was between 7.0 and 11.8 mg/kg body weight. Neither vomiting nor regurgitation was observed in any Credelio™ treated animal during the clinical assessment after treatment. No adverse events related to the oral administration of Credelio™ chewable tablets for cats were observed in any cat at any time during the study.

### Efficacy parameters

The primary efficacy criterion in this study was the total number of viable mites (sum of larvae, nymphs and adults in right and left ears) assessed following ear flushing on day 28. Secondary efficacy criteria were the reduction of live mite count score and the improvement of debris score, both assessed during otoscopic examination on days 14 and 28.

The individual mite count scores (days −2, 14 and 28) as well as the mite counts after ear flushing on day 28 are listed in Table 1. Credelio™ treatment significantly reduced ear mite infestation in cats following a single treatment. On day 28 after ear flushing, the arithmetic mean live *O. cynotis* mite count (both ears, larvae, nymphs and adults) in the Credelio™ treated group was 1, compared to 382 in the control group, thus resulting in a significant (*P* = 0.0008) reduction of mites and an efficacy of 99.7% (arithmetic mean) (Table 2).

**Table 2:** Total live mite count data and corresponding efficacy (%) of Credelio™ administered once orally to cats against experimentally induced infestations with *Otodectes* *cynotis* at 28 days after treatment

Study group and efficacy	Range of total live mite counts^a^ day 28	Arithmetic mean	Geometric mean
Credelio™ group	0–4	1.0	0.6
Control group	6–1502	382.0	164.5
Efficacy (%)		99.7 (*P* = 0.0008^b^)	99.6 (*P* = 0.0002^c^)

^a^Total live mite counts = sum of adults, nymphs and larvae recorded following ear flushing of both right and left ears

^b^Wilcoxon-Mann-Whitney test (*Z* = 3.3605)

^c^Mixed analysis of variance (ANOVA) with sex and treatment group as fixed effects and block as random effect (*t*_(7)_ = 7.23)

**Fig. 1:**
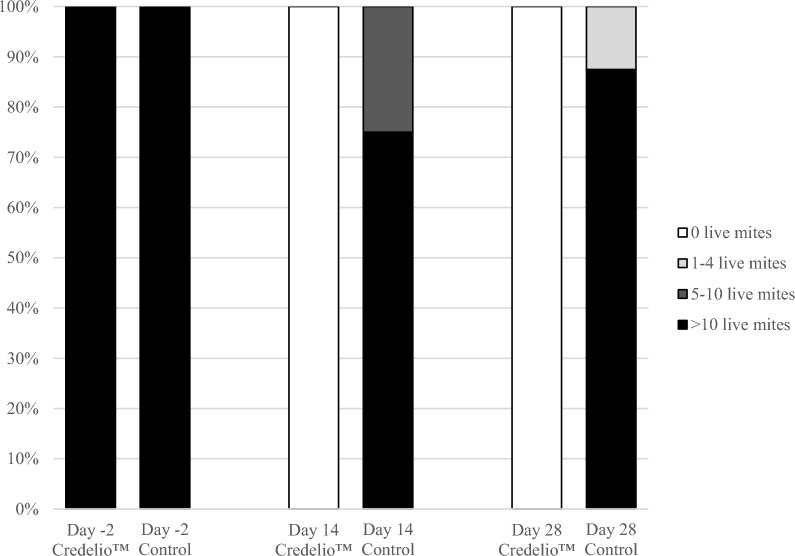
Percentage of cats with live mites observed during otoscopic examinations pre-treatment on day −2 and after treatment on days 14 and 28

Cats treated orally with Credelio™ had no mites visible (score 0) during otoscopic examination at either day 14 or day 28 after treatment (Table 1; Fig. 1), demonstrating 100% reduction of live mite count scores and being significantly different to the control group on both study days (day 14 [*P* = 0.0027; CMH test, *χ*^2^ = 9.0000, *df* = 1], day 28 [*P* = 0.0006; CMH test, *χ*^2^ = 11.6667, *df* = 1]).

**Fig. 2:**
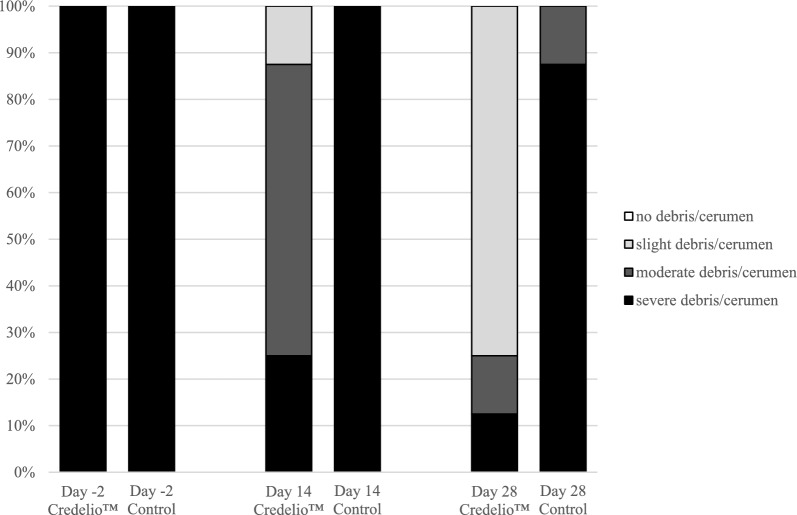
Percentage of cats with debris/cerumen observed during otoscopic examinations pre-treatment on day -2 and after treatment on days 14 and 28

Credelio™-treated cats showed an improvement in the amount of otic debris/cerumen by day 28 after treatment (Fig. 2) compared to baseline on day -2. By day 14, 75% of the Credelio™-treated cats had improved from the worst score of 3 (severe debris/cerumen) on day -2, and by day 28 this had increased to 87.5% of Credelio™-treated cats. In the control group cats there was no improvement by day 14 and only 12.5% of the control group cats had improved by day 28, from a score of 3 on day -2. Improvement of the debris score was significantly different between the groups, both on day 14 (*P* = 0.0027; CMH test, *χ*^2^ = 9.0000, *df* = 1) and day 28 (*P* = 0.0037; CMH test, *χ*^2^ = 8.4375, *df* = 1).

## Discussion

A single oral administration of lotilaner (Credelio™ chewable tablets for cats) at a dose between 7.0 and 11.8 mg/kg body weight (i.e. at the lower end of the recommended dose range for cats [6–24 mg/kg]) was highly efficacious in treating cats experimentally infested with *O. cynotis.* Based on quantitative live mite counts 28 days after treatment, the efficacy (geometric mean) of a single Credelio™ administration was 99.6%, with a significant reduction in the total live mite counts in the Credelio™ treated group compared to a sham-dosed control group.

In total, different developmental stages of ear mites were recovered after ear flushing on day 28 in three of eight treated cats, with a geometric mean of 0.6, while eight of eight control group cats were positive for ear mite stages on day 28, with a geometric mean of 164.5. In contrast to these ear flushing results, otoscopic examination during the study period on day 14 and 28 did not reveal any ear mite stages in the treated group. Thus, in this study, ear flushing and subsequent specimen examination showed a higher sensitivity in comparison to a pure otoscopic examination. This difference in methodological sensitivity has also been documented in a study comparing conventional handheld otoscopy with curette sampling or cotton-tipped swabbing [56]. In the current study, the method of ear flushing could only be applied at the end of the study due to the nature of removing ideally all debris, cerumen and also mites by flushing.

Considering the clinical signs in the ear mite-infested animals, the 28-day period in the reported study between treatment and the subsequent assessment of clinical signs was too short to allow a complete regression and resolution of ear mite-associated presence of debris and cerumen. This observation is not unexpected, as it is known that the disappearance of otocariosis signs may take longer and that some degree of clinical signs may still be present in cats for several days after testing negative for ear mites [13]. Nevertheless, the immediate decrease in the number of mites from the external ear canals, as assessed by zero live mite counts during otoscopic examination 14 and 28 days after treatment, was accompanied by a rapid improvement in the clinical signs of otocariosis in the Credelio™ treated group (improvement in 75% of the study population at day 14 and in 87.5% at day 28).

Due to the highly contagious character of otodectic mange, prevention of a disease recurrence strongly depends on the miticidal activity of the corresponding treatment [57]. However, despite an effective treatment of the host animal, the living place, cage and grooming equipment used by and on infested animals can also be a source of mites and thus of reinfestation [9], as mites can survive off the host for 12 days [14]. These sources of reinfestation should be taken into consideration.

Lotilaner’s high miticidal efficacy against *O. cynotis* supports previously published studies that have all reported excellent acaricidal efficacy of isoxazolines against ear mites in cats. In one study, a chewable formulation containing afoxolaner and registered for use in dogs (2.5 mg/kg; NexGard®; Boehringer Ingelheim, Ingelheim, Germany) was reported to be 100% effective for the treatment of otodectic mange in cats, based upon the absence of mites upon otoscopy 48 h after oral administration and weekly thereafter [58]. For the topically-applied isoxazolines registered for use in cats, fluralaner (40 mg/kg; Bravecto®; Merck & Co., Inc., Rahway, NJ, USA), esafoxolaner (1.44 mg/kg in combination with eprinofmectin 0.48 mg/kg and praziquantel 10 mg/kg; NexGard® COMBO; Boehringer Ingelheim) and sarolaner (1 mg/kg in combination with selamectin 6 mg/kg; Revolution® Plus; Zoetis) resulted in > 99–100%, > 97% and > 99% efficacy against experimental infestations of *O. cynotis*, respectively, based on live mite counts after ear flushing 1 month after treatment [47, 48, 50, 52]. A topical formulation of tigolaner, a molecule within the bispyrazole class of ectoparasiticides (14.4 mg/kg in combination with emodepside 3 mg/kg and praziquantel 12 mg/kg; Felpreva®; Vetoquinol S.A., Lure, France) has also shown to be ≥ 99.6% effective against experimental *O. cynotis* infestations in cats, based on live mite counts after ear flushing 28 days after treatment [57].

Until the introduction of isoxazolines onto the market, treatment of ear mites in cats relied heavily upon topically applied endectocides consisting either of one (selamectin) or a combination of two (moxidectin and imidacloprid) pharmaceutical ingredients applied either as a single spot-on application or as two administrations 1 month apart [43–46]. There have also been a number of otic medications used for treatment of otodectic mange, some containing miticidal agents, others thought to work by smothering the mite. The main disadvantages of these treatments are the need for long-term treatment (mostly daily for 21–30 days), patient compliance and the possibility of reinfestation from the environment [59]. An advantage of otic medications that include antimicrobial agents is the concurrent treatment of secondary fungal or bacterial otitis, which is a common sequela to otocariosis [59]. In earlier topical therapies involving acaricidals directly applied into the ear canals, active ingredients had limited residual activity and again required daily application for 3 weeks to ensure that all eggs hatched and emerging larvae were exposed to the drug [9, 59].

It is widely acknowledged that owner compliance with intensive treatment regimens is poor, making an easy-to-give, highly effective, monthly treatment for otocariosis appealing to cat owners and their veterinary teams. In addition to owner’s convenience, animal’s temperament should also be considered when evaluating treatment options in respect to frequent applications such as ear drops multiple times per day [9]. Lotilaner in the form of Credelio™ chewable tablets for cats are small and palatable tablets [35], making them easy to administer to cats from 8 weeks of age. This characteristic, taken together with a single application being safe and well tolerated and possessing a high miticidal efficacy (99.6%) in cats infested with *O. cynotis*, without any supportive measures, such as regular cleaning of the ears, highlights the potential of lotilaner for the convenient and effective treatment of ear mite infestations in cats, not only by eliminating ear mite infestation from the ear canal, but also by interrupting the life cycle of *O. cynotis* mites in almost all cats after a single application, in addition to killing fleas and ticks for one month.

## Conclusions

The study reported here demonstrates that a single oral dose of Credelio™ chewable tablets for cats (lotilaner) was well tolerated, safe and highly effective for the treatment of *O. cynotis* infestations in cats. The single oral lotilaner dose was highly efficacious (99.6%) in eliminating ear mite infestations and greatly improved clinical signs of otocariosis during the 28-day study period.

## Data Availability

The dataset summarizing and supporting the conclusions of this article are included within the article. Due to commercial confidentiality of the research, data not included in the manuscript can only be made available to bonafide researchers subject to a fully executed non-disclosure agreement.

## Abbreviations

CMH test: Cochran-Mantel–Haenszel test
CVMP: Committee for Veterinary Medicinal Products
VICH: Veterinary International Conference on Harmonization
